# Spatiotemporal noise characterization for chirped-pulse amplification systems

**DOI:** 10.1038/ncomms7192

**Published:** 2015-02-04

**Authors:** Jingui Ma, Peng Yuan, Jing Wang, Yongzhi Wang, Guoqiang Xie, Heyuan Zhu, Liejia Qian

**Affiliations:** 1 Key Laboratory for Laser Plasmas (Ministry of Education), Department of Physics and Astronomy, IFSA Collaborative Innovation Center, Shanghai Jiao Tong University, Shanghai 200240, China; 2 Shanghai Engineering Research Center of Ultra-precision Optical Manufacturing, Department of Optical Science and Engineering, Fudan University, Shanghai 200433, China

## Abstract

Optical noise, the core of the pulse-contrast challenge for ultra-high peak power femtosecond lasers, exhibits spatiotemporal (ST) coupling induced by angular dispersion. Full characterization of such ST noise requires two-dimensional measurements in the ST domain. Thus far, all noise measurements have been made only in the temporal domain. Here we report the experimental characterization of the ST noise, which is made feasible by extending cross-correlation from the temporal domain to the ST domain. We experimentally demonstrate that the ST noise originates from the optical surface imperfections in the pulse stretcher/compressor and exhibits a linear ST coupling in the far-field plane. The contrast on the far-field axis, underestimated in the conventional measurements, is further improved by avoiding the far-field optics in the stretcher. These results enhance our understanding of the pulse contrast with respect to its ST-coupling nature and pave the way toward the design of high-contrast ultra-high peak power lasers.

## Abstract (web summary)

High-field physics experiments are often plagued by noise in the driving laser, which remains poorly characterized. Ma *et al*. study the noise in a pulse stretcher and compressor system in the spatiotemporal domain and find that noise from the stretcher elements governs the pulse contrast at the laser focus.

## Graphical abstract

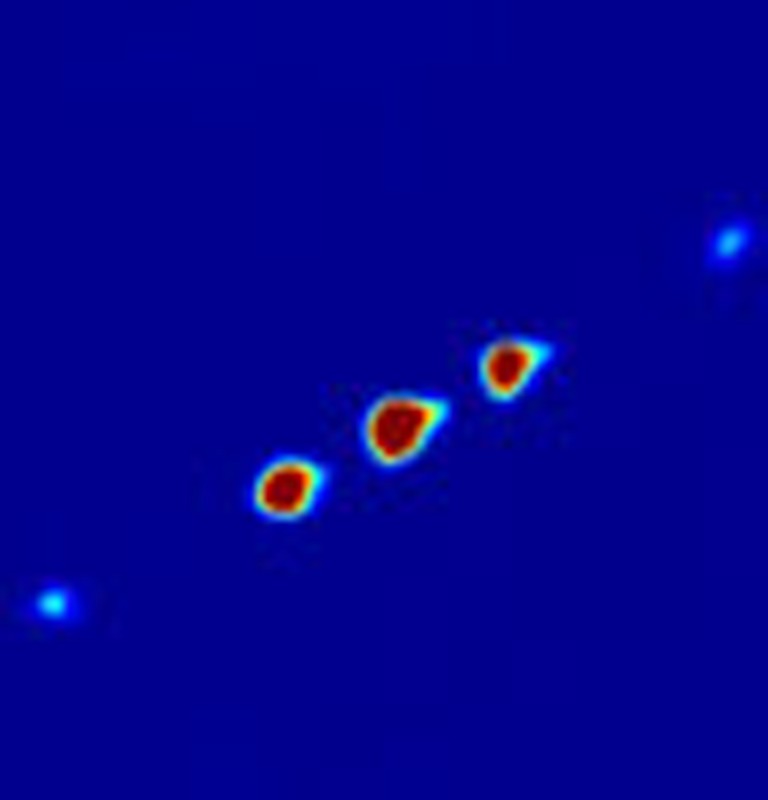

In the past decade, significant breakthroughs in extreme high-field physics have been made possible by the development of ultra-high peak power lasers. One recent example of such a breakthrough is the plasma acceleration of particles^1^,^2^,^3^,^4^. The fundamental limits on such experiments are often governed by the noise in laser systems. Pulse contrast, the intensity ratio of the laser pulse peak to its noise background, is routinely improved by non-linearly filtering out the noise of the seed pulse before chirped-pulse amplification (CPA)^5^,^6^. At present, a contrast level of 10^8^–10^10^, in the time scale of several to hundreds of picoseconds beyond the main pulse can be achieved for femtosecond lasers with peak powers of hundreds of terawatts to petawatts^7^,^8^,^9^. The quest for higher powers and better contrast drives attempts to reduce the other two types of noise produced in CPA, that is, the noise from laser amplifiers and the noise from pulse stretchers/compressors. However, there have been few remarkable achievements in this direction to date.

In this study, we report the characterization of noise produced by pulse stretchers and compressors. Because of spatiospectral coupling by angular dispersion^10^,^11^, the surface imperfections of an optical element in a stretcher or compressor will be imprinted onto the pulse spectrum, resulting in spatiotemporally coupled noise after pulse compression and beam focusing^12^. Such spatiotemporal (ST) coupling can be better illustrated, for example, by placing a one-dimensional (1D) periodic amplitude mask in the pulse stretcher or compressor. Assuming that the mask has a period of Λ along the spectral dispersion direction with a spatiospectral coupling factor (that is, the inverse of the spatial chirp) of *δ* (*δ*=d*x*/d*ω*), the laser-intensity distributions after pulse compression can be expressed as^13^,


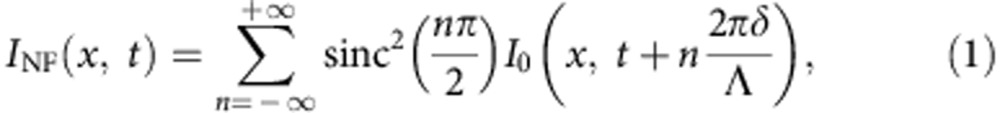



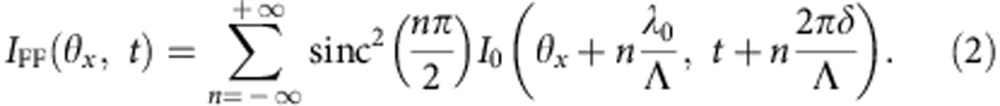


The near-field distribution (*I*_NF_) displays a series of space-independent side pulses (odd numbers only) around the main pulse (*n=*0), and the far-field case (*I*_FF_) clearly reveals an ST-coupling effect. The locations of the side pulses depend on both the angular and temporal coordinates and lie along a slope of *α*=2*πδ/λ*_0_. In the same manner, the noise produced by elements in the pulse stretcher and/or compressor will exhibit ST-coupling characteristics similar to those of the above far-field pulsed beam. At this point, we anticipate that the ST noise will widely exist in processes involving angular dispersion, such as achromatic phase matching^14^, ultrafast pulse shaping^15^ and frequency domain optical parametric amplification^16^. Thus, in the fields of ultrafast optics and ultra-high peak power lasers, a major experimental challenge is to directly and quantitatively analyse the ST noise over an angular range around the far-field axis.

Noise or contrast measurement requires both a large temporal window and a high dynamic range. The currently available ST measurement techniques for laser pulses^17^,^18^,^19^, such as spatially encoded arrangement for temporal analysis by dispersing a pair of light E-fields (SEA TADPOLE), are not appropriate for characterizing ST noise due to their limited dynamic ranges (<10^5^). Recently, cross-correlation techniques, in either time-scanning or single-shot mode, have been developed for high dynamic range measurements of pulse contrast^20^,^21^,^22^,^23^,^24^,^25^,^26^,^27^,^28^,^29^. These measurements, which are commonly performed in the near field and are only temporally resolved, have been successful in characterizing noise without ST coupling, such as that produced in laser amplifiers^30^,^31^,^32^. Techniques capable of both temporal and spatial resolution for the characterization of ST noise are desired^12^,^33^, especially for assessments in high-field physics in which the experimental target is located in the far field.

Here we extend cross-correlation from the temporal domain to the ST domain to investigate the ST noise produced in pulse stretchers and compressors. The two-dimensional (2D) measurement in the *θ*_*x*_*-t* domain that can be achieved using the ST cross-correlator (STCC) even enables the identification of the origins of the ST noise, which would otherwise not be possible. For the situation in which the noise from the laser amplifier is well eliminated, we experimentally demonstrate that the far-field on-axis pulse contrast, suitable for high-field physics experiments, is dominated by the noise from the far-field optical elements that are typically used in the pulse stretcher (that is, convex mirrors). The results presented in this study should be valuable for the construction of high-contrast ultra-high peak power lasers and their applications in high-field physics experiments.

## Results

### Spatiotemporal cross-correlator

**Figure 1:**
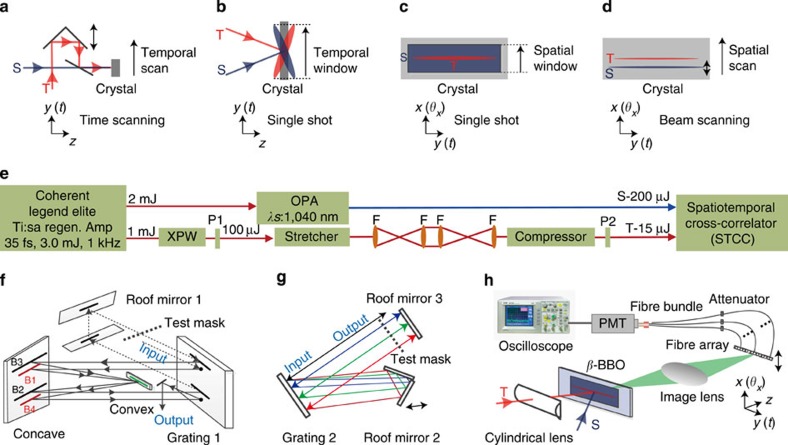
Experimental setup. (**a**,**b**) Schematic diagrams of (**a**) time-scanning and (**b**) single-shot cross-correlations in the temporal domain. (**c**,**d**) Schematic diagrams of (**c**) single-shot and (**d**) beam-scanning cross-correlations in the spatiotemporal domains. (**e**) Block diagram of the experimental setup. P1 and P2 are periscopes. (**f**) Side view of the Öffner stretcher. B1 through B4 are the four beam locations on the concave mirror. (**g**) Top view of the compressor. (**h**) Diagram of the STCC. T and S are the under-test pulse and sampling pulse, respectively.

The proposed STCC consists of three key elementary components: an ST cross-correlation component based on sum-frequency generation (SFG), a 1D detection system that can be translated in the direction of ST coupling and a sampling laser. In a conventional cross-correlator, the temporal window is realized by an optical delay line in the scanning mode (Fig. 1a) or by space-to-time encoding via beams intersecting in the single-shot mode (Fig. 1b). Typical commercially available cross-correlators, for example, Sequoia (Amplitude Technologies, France), have no spatial resolving capability. The proposed STCC is an extension of the conventional single-shot apparatus, allowing 2D [*y* (*t*) and *x* (*θ*_*x*_)] mapping of the pulse profile in the far field to be realized in a single-shot manner if a rectangular sampling beam of sufficient size and a 2D detector are adopted (Fig. 1c). However, currently available 2D detectors, for example, charge-coupled devices (CCDs), offer a limited sensitivity and dynamic range. In our previous studies, we demonstrated a highly sensitive detection system based on a combination of a fibre array and photomultiplier tube (PMT)^29^, as illustrated in Fig. 1h, which can support a dynamic range of >10^10^ and 100 sampling points. In this experimental study, we used a 1D fibre array that can be translated along the *θ*_*x*_ direction as an alternative to a 2D detector. For a certain far-field angle, detection can still be accomplished in a single-shot manner. Several measurements, row by row, are needed to obtain a complete picture of the ST noise in the far field. We refer to this mode of operation as the detector-scanning mode. We are also able to focus the sampling beam in 1D and scan it across the beam under test (Fig. 1d), with signal tracing via a synchronous 1D fibre-array translation, which is more advantageous if the pulse energies are insufficient. We call this mode of operation beam-scanning mode. These awkward alternative modes (that is, the detector-scanning or beam-scanning modes) are necessary because of the lack of appropriate detection devices and/or insufficient pulse energies, not because of fundamental limitations. A true single-shot 2D measurement of the ST noise would be possible if a 2D fibre array was used and also sufficient input energy was available.

As is done with a conventional cross-correlator, sampling pulses for the STCC can be generated via second-harmonic generation^20^,^21^,^22^,^23^,^24^,^25^,^26^ or optical parametric amplification (OPA)^27^,^29^, either of which will be clean in both the temporal and spatial domains. The STCC is illustrated in Fig. 1h. The under-test laser, with a round beam size of 13 mm (the diameter containing 86.5% of the pulse energy) and a wavelength of 800 nm, was focused in 1D to a size of ~100 μm in the *x* (*θ*_*x*_) direction by a cylindrical lens (*f*=50 cm). In the detector-scanning mode, a sampling laser at 1,040 nm from a femtosecond OPA (see Methods for details) was shaped to a beam size of 4 mm (*x*) × 15 mm (*y*). In the beam-scanning mode, the sampling laser was cylindrically focused to a beam size of ~100 μm (*x*) × 15 mm (*y*). In both modes, SFG signals were produced by a 3-mm thick type I β-BBO crystal cut at 78°, which was located at the focus of the under-test laser. The intersection angle between the interacting beams was 38.8°, which could support a temporal window of 36 ps cm^−1^. The SFG signal at 452 nm was imaged onto a 1D fibre array (13 mm wide) by a spherical lens (*f*=20 cm). The detection system consists of 100 ultraviolet fibres (Ceram Optec, Germany) with incremental lengths from 3 to 102 m (with a length increment between adjacent fibres of 1 m) and a highly sensitive PMT (H6780-04, Hamamatsu, Japan). The spatial and temporal resolutions of the STCC are approximately 0.2 mrad and 0.5 ps, as determined by the diffraction-limited beam divergence and the number of fibres used in the detection system, respectively.

### Verification of the operation of the STCC

**Figure 2:**
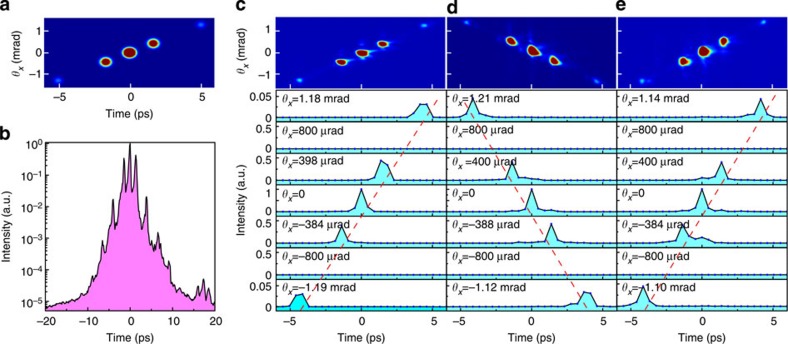
Verification of the STCC. (**a**) Calculated far-field distributions of side pulses induced by the test mask placed in the compressor. (**b**) Near-field pulse profile measured using a time-scanning cross-correlator. (**c**–**e**) Experimental results for the side pulses induced with the mask placed (**c**) in the compressor, (**d**) in the stretcher and (**e**) in the stretcher with only one image-relay telescope between the stretcher and compressor. In (**c**–**e**), the upper panels are the recorded CCD images, and the lower panels display the four-side pulses and the main pulse (*θ*_*x*_=0). The dashed line in each panel, determined by the locations of side pulses, highlights the ST slope.

The measurement of the ST structures determined by equation (2) was used as a representative task to verify the proper operation of the STCC. The theoretical formula for the *n*th-order induced side pulse predicts an intensity of *I*_*n*_*=*(2/*nπ*)^2^*I*_0_ and an ST location of (*θ*_*n*_*=*−*nλ*_0_/Λ, *T*_*n*_=−2*πnδ*/Λ). This type of known ST pulse structure was measured using the STCC with a large sampling beam (Fig. 1c). In the experiments, the cross-polarized wave generation (XPW, see Methods for details) technique was adopted to clean the femtosecond pulses produced by a Ti:sapphire regenerative amplifier^5^. The output was sent to a home-built Öffner stretcher^34^ and compressor, as depicted in Fig. 1f,g (see Methods for detailed parameters), with an amplitude mask (Λ=2 mm) inserted between roof mirror 3 and grating 2 in the compressor. The output from the stretcher and compressor was then focused cylindrically and served as the under-test pulse. The numerically calculated ST distribution of the under-test pulse is presented in Fig. 2a. For the parameters of this experiment, the theoretically predicted position of the *n*th-order side pulse is (*θ*_*n*_=−0.4 × *n* mrad, *T*_*n*_=−1.68 × *n* ps).

As expected, the conventional time-scanning cross-correlation trace, presented in Fig. 2b, cannot reveal the ST-coupling characteristics because of spatial smearing. The characterization of such an ST-coupling effect requires the use of an STCC. Although not ideal in terms of its sensitivity and dynamic range, a CCD camera is still quite appropriate for acquiring correlation images and determining the positions of the side pulses. A CCD-captured single-shot 2D cross-correlation image is presented in Fig. 2c, and the image corresponds well with the calculated distribution (Fig. 2a). The fibre array-based detection system (that is, the detector-scanning mode) was then used to record a set of row signals at several far-field angles, and the results are presented in Fig. 2c. The measured ST locations and intensities of the four-side pulses (*n*=±1 and ±3) correspond well with the theoretical values given above. For instance, the first-order (*n*=1) side pulse displays an intensity of 0.38 at a location of (−384 μrad, −1.4 ps). In addition, the side pulses line up in the *θ*_*x*_–*t* plane as predicted. As indicated by equation (2), the line slope *α* is determined by the spatiospectral coupling factor *δ* and the central wavelength *λ*_0_. We may change the sign of the slope by either moving the mask from the compressor to the stretcher (Fig. 2d) or by adding one image-relay telescope between the stretcher and the compressor (Fig. 2e). This implies that the slope and its sign can be used to identify the magnitudes and origins of different ST structures or noise components.

### Measurement and identification of ST noise

**Figure 3:**
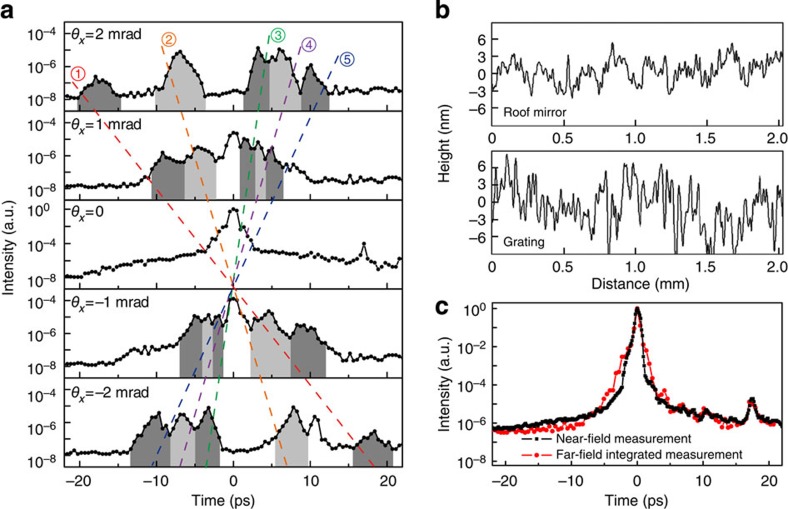
Measurements of the ST noise. (**a**) Measured ST noise components produced in the stretcher and compressor at five far-field angles. Each shaded region represents a specific noise component. Five dashed lines, indicating the ST slopes, correspond to the five groups of ST noise shown in Table 1. (**b**) Measured surface roughnesses of the roof mirror and grating in the compressor. (**c**) Near-field pulse profile (the black curve with square symbols) and far-field pulse profile integrated over an angular window of |*θ*_*x*_|≤4 mrad (the red curve with circle symbols). See Methods for details.

The STCC in the beam-scanning mode was applied to measure the ST noise produced in the pulse stretcher and compressor. To ensure that this ST noise dominated in the under-test pulse, the output pulses from the Ti:sapphire regenerative amplifier were cleaned using the XPW technique before being sent to the tested pulse stretcher and compressor. Noise measurements were performed at five far-field locations of *θ*_*x*_=0, ±1 and ±2 mrad, and the results are summarized in Fig. 3a. All five correlation curves exhibit a similar background of approximately 10^−8^ with respect to the pulse peak at *θ*_*x*_=0, indicating a dynamic range of approximately 10^8^ for the STCC measurements.

Based on their ST slopes, the measured ST noise components can be divided into five sets, numbered from 1 to 5 in Fig. 3a. Each set corresponds to a certain value of *α* that can be traced back to its source optics, as shown in Table 1. These ST noise components originate from the Au-coated surfaces of the roof mirrors, concave mirror and gratings used in the stretcher and/or compressor. The ST noise components from the grating and vertical roof mirror in the stretcher (or compressor) overlap in the ST domain because they share the same value of *δ*. Thus, the measured ST noise numbered as 2 (4) contains contributions from both the grating and the vertical roof mirror in the stretcher (compressor). The noise components from all optics in the compressor, that is, the grating and roof mirror, correspond to positive ST slopes, whereas those from the stretcher may correspond to either negative or positive ST slopes. Because the image-relay telescope changes the sign of the ST slope as the dispersed beam passes through it, the concave mirror in the stretcher produces two ST noise components with opposite slope signs (1 and 5 in Fig. 3a). Unlike the other optics, the convex mirror produces conventional noise without ST coupling because it is located at the beam focus. Noise introduced by the optics located in the far-field will be discussed in detail in the next section.

All optics used in the stretcher and in the compressor were inspected using a surface profilometer (PGI Dimension, Taylor Hobson, UK). Typical surface profiles of the roof mirrors and gratings are presented in Fig. 3b. As suggested by theoretical analysis^12^, the ST noise is proportional to the square of the surface roughness. In the experiments, we confirmed a strong relation between the noise strength and surface roughness. For instance, all measured surfaces exhibited quite similar roughnesses of 3–5 nm at a spatial frequency of ~2.5 mm^−1^, consistent with the experimental observations of comparable noise at *θ*_*x*_=±1 mrad. At this point, it is necessary to compare the measurements acquired using the STCC with those acquired with the conventional cross-correlator. All ST structures become smeared over the *x* dimension when the conventional cross-correlator is used (the black curve in Fig. 3c), which is equivalent to the angularly integrated result of the STCC measurements (the red curve in Fig. 3c). As a result, the indicated noise level (~5 × 10^−7^) is unavoidably overestimated compared with the more realistic and more accurate results (~10^−8^) provided by the STCC. Such a misleading result is detrimental in high-field physics experiments and STCC-based measurements are necessary.

**Figure 4:**
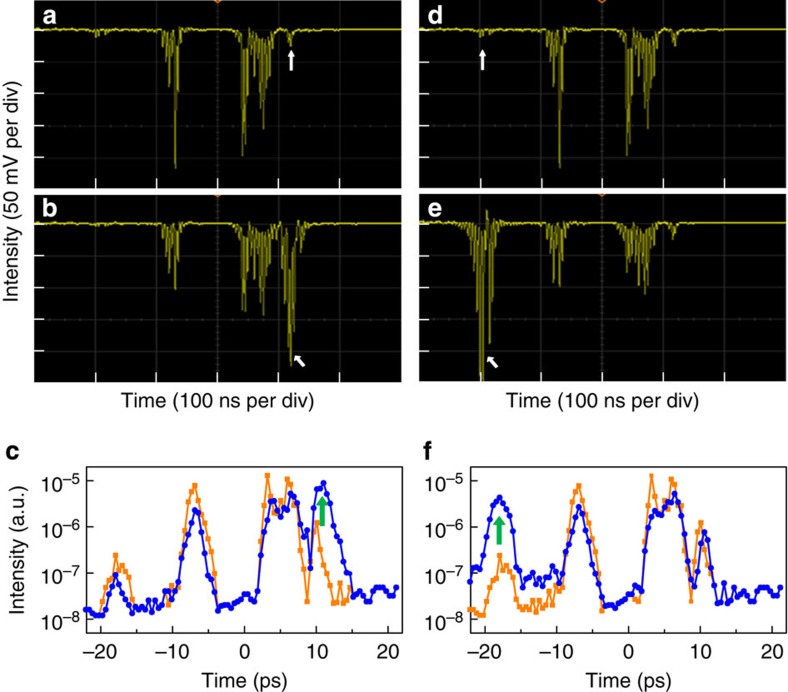
Identification of the ST noise components. (**a**,**d**) Oscilloscope display of the ST noise components at *θ*_*x*_=2 mrad. (**b**,**e**) Oscilloscope display of the ST noise components at *θ*_*x*_=2 mrad with lens–tissue clipping of beam (**b**) B1 and (**e**) B2. (**c**,**f**) Measured far-field noise components corresponding to (**c**) traces in panels (**a**,**b**) and (**f**) traces in panels (**d**,**e**). The blue curves with circle symbols (the orange curves with square symbols) represent the results with (without) a lens tissue. Each white arrow in (**a**,**b**,**d**,**e**) indicates the noise component under identification, while each green arrow in (**c**,**f**) highlights the increase of the specific noise component.

The 2D-mapping capability of the STCC allows for the *in situ* identification of the individual origins of the ST noise components. This identification can be accomplished simply by placing a lens tissue in front of the surface of a specific optical element, partially clipping into the laser beam; the corresponding noise will then typically be enhanced by more than one order of magnitude. This selective noise enhancement can be monitored in real time using an oscilloscope. For example, Fig. 4 presents the results of such noise enhancement for the concave mirror in the Öffner stretcher. When a lens tissue was placed in the beam path of the first or fourth pass (B1 or B4, respectively, in Fig. 1f), the selective enhancement occurred on the right-hand side (Fig. 4a–c). Similarly, the enhancement occurred at the left-hand side (Fig. 4d–f) when beam B2 or B3 was clipped. Thus, the concave mirror indeed induced two components of ST noise with opposite slope signs (1 and 5 in Fig. 3a) because of the effect of the image-relay telescope, composed of concave and convex mirrors. Using this method, we experimentally confirmed that the noise components from the grating and vertical roof mirror in the stretcher (or compressor) would overlap in the ST domain.

### Far-field on-axis pulse contrast

The previous sections demonstrated that surface imperfections of optics located in the near field in the stretcher and compressor may significantly degrade the far-field pulse contrast, especially in off-axis positions (*θ*_*x*_≠0). For optics with surface roughnesses of 3−5 nm, the typical off-axis pulse contrast was 10^5^−10^6^, depending on the ST location (Fig. 3a). On the axis (*θ*_*x*_*=*0), however, this ST noise will affect the pulse contrast predominantly within a limited range of |*t*|≤*αθ*_F_/2 around the main pulse (where *θ*_F_ is the acceptance angle of the fibre, 0.2 mrad).

**Figure 5:**
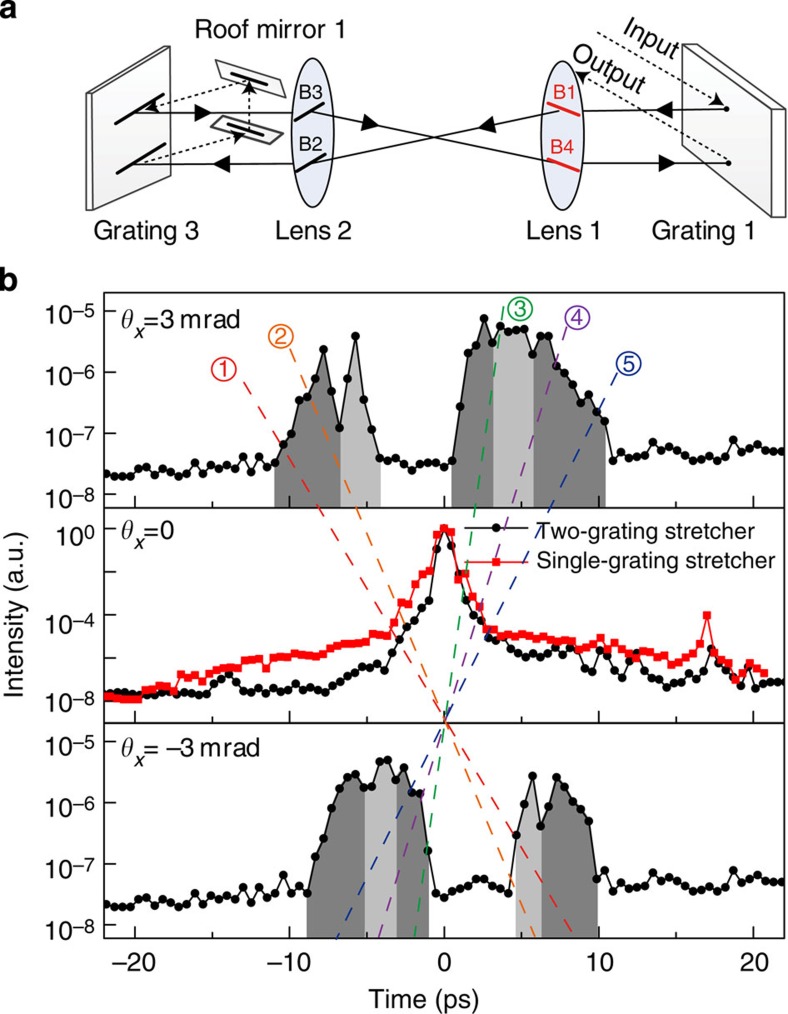
Measurements of the ST noise components using a two grating-based stretcher. (**a**) Side view of the two grating-based stretcher. (**b**) Measured ST noise components. The black curve with circle symbols (the red curve with square symbols) in the panel corresponding to *θ*_*x*_=0 represents the measurement for the two grating-based (single-grating) stretcher. Each shaded region represents a specific noise component, and the five dashed lines highlight the ST slopes.

In contrast, the on-axis pulse contrast (Fig. 3a, *θ*_*x*_*=*0) was governed by the noise from the convex mirror in the Öffner stretcher. Without ST coupling, the noise from the convex mirror will be spatially confined around *θ*_*x*_*=*0 because the convex mirror is located at the beam focus. Avoiding the use of a convex mirror in the stretcher may greatly improve the far-field on-axis pulse contrast. To demonstrate this possibility experimentally, we replaced the Öffner stretcher with a two grating-based stretcher without any far-field optics (Fig. 5a). In this case, the measurements acquired using the STCC also revealed five ST noise components (Fig. 5b). However, the on-axis pulse contrast was significantly improved (black curve with circle symbols at *θ*_*x*_*=*0 in Fig. 5b) to ~100 times higher than that achieved using the Öffner stretcher. A design for a cylindrical Öffner stretcher was proposed to avoid beam focusing in the plane of spatiospectral coupling^35^. The experimental results presented here may support this type of stretcher design.

## Discussion

High-power ultrashort pulses, after ideal compression, are not coupled between time and space. However, this is not the case for laser noise; instead, laser noise is naturally ST coupled because of surface imperfections in the CPA apparatus. Noise characterization strongly requires a 2D measurement in the ST domain, a large temporal window and a high dynamic range. Using the STCC demonstrated here, we measured the spatially resolved pulse contrast at and around the far-field axis and revealed the natures of the ST-coupled noise components produced in the pulse stretcher and compressor. It is now important to summarize the full ST noise distribution using a single pulse-contrast value. Because most applications of ultra-high peak power lasers require beam focusing, we suggest that the pulse contrast in this case can be defined on the far-field axis. As experimentally demonstrated, the far-field on-axis pulse contrast is markedly different from the near-field pulse contrast. Moreover, the far-field off-axis noise components are anticipated to have only a slight effect on high-field physics experiments conducted at the beam focus. Under the assumption of a certain noise at an ST position of (*θ*_*x*_, *t*), the distance between this point and the beam focus is *ct*(1+*θ*_*x*_^2^)^1/2^, which results in a light time delay between the two points that is always greater than *t* (*c* is the speed of light). Thus, the preplasma produced by this off-axis noise will not disturb on-axis experiments regardless of the noise location. In addition, the off-axis noise can be well eliminated when spatial filters are adopted in CPA laser systems, as suggested by our measurements in Figs 3 and 5. Therefore, we conclude that only the on-axis pulse contrast is relevant in high-field physics experiments.

In conclusion, we demonstrated the ability to measure and identify the ST noise components produced in pulse stretchers and compressors. We found experimentally that the ST noise is transferred from the optical surface imperfections. Significant enhancement of the on-axis pulse contrast can be achieved by avoiding the far-field optics in the stretcher. By extending cross-correlation from the temporal domain to the ST domain and adopting a sensitive fibre-array detection system, we demonstrated an STCC with a dynamic range of 10^8^, a temporal window of 50 ps and an angular range of 6 mrad. At this point, it is important to address the scalability of this method to a higher dynamic range. In our experiments, the energies of the sampling and under-test pulses were only 200 and 15 μJ, respectively. Because the generated SFG signal peak is proportional to the product of the energies of the interacting pulses, a higher dynamic range, for example, >10^10^, would be possible if the energies of both pulses were to be increased to the 1 mJ level. Such a device is promising for the construction and optimization of high-contrast ultra-high power laser systems. In addition to this application, this ultrasensitive cross-correlation architecture may also be applied to ultrafast nonlinear optics^36^,^37^, ST-resolved spectroscopy^38^ and, in general, the control of ST focusing through inhomogeneous media in microscopy and nanosurgery^39^,^40^.

## Methods

### Experimental setup

The experimental layout is schematically illustrated in Fig. 1e. The laser source is a femtosecond Ti:sapphire regenerative amplifier with a central wavelength of 800 nm. One portion of its output (2 mJ) is used to pump an LiNbO_3_-based OPA system to generate a clean sampling pulse at 1,040 nm (200 μJ), whereas the remainder (1 mJ), after passing sequentially through an XPW, a stretcher and a compressor, is used as the under-test pulse (15 μJ). The femtosecond OPA system consists of a double-pass optical parametric generation stage and a single-pass OPA stage. Two 14- and 8-mm thick uncoated LiNbO_3_ crystals, both cut at 45°, are used in the two stages. The XPW consists of a 10-mm thick BaF_2_ crystal, two Glan polarizers (GL10-B, Thorlabs) and two convex lenses both of which have a focal length of 1 m. The Öffner stretcher consists of a concave mirror (*R*=2 m), a convex mirror (*R*=−1 m), a vertical roof mirror and a diffraction grating. The grating, with 1,480 lines mm^−1^ (Horiba Jobin Yvon, France), is located 152.5 cm away from the concave mirror. The compressor consists of a grating of the same type as that used in the stretcher, a vertical roof mirror and a horizontal roof mirror. The equivalent dispersion length of the compressor is 101.5 cm. The incident angles at the gratings in the stretcher and compressor are both set to 45°. The two grating-based stretcher uses two gratings of the same type as above, two convex lenses (*f*=75 cm) and a vertical roof mirror and the distance between the first grating and the first convex lens is set to 35 cm.

### Angular integration of STCC measurements

The angularly integrated contrast in the far-field (the red curve in Fig. 3c) was measured by slightly altering the setup of the STCC. In such an experiment, a cylindrical lens (*f*=3 cm) was added before the fibre array (Fig. 1h), which couples all the correlating signals within an angular window (|*θ*_*x*_|≤4 mrad) to the fibre channels and hence to the detector.

### Near-field pulse-contrast measurement

The near-field pulse contrast (the black curve in Fig. 3c) was measured using a time-scanning cross-correlator (Fig. 1a). The sampling pulse was also the 1,040-nm pulse generated by the femtosecond OPA. The sampling and under-test pulses were collinearly incident on a 2-mm thick type I β−BBO crystal to generate the SFG correlation signal. The same PMT used in the STCC was applied to detect the correlation signal.

### Spatiospectral coupling factor *δ* and ST slope *α*

Here we provide a formula to relate *δ* and *α* to the parameters of the pulse stretcher and compressor. The angular dispersion of the grating is *β*=d*σ*/d*ω*=*λ*_0_^2^/(2*πcd*cos*θ*_0_), where *σ* and *d* are the diffraction angle and grating constant, respectively^11^. Using the simple relation between the transverse deviation and the propagation distance (d*x*=*L*·d*σ*), the spatiospectral coupling factor *δ* can be found to be *δ*=d*x*/d*ω*=*βL*. According to equation (2), the ST noise slope *α* is determined by the value of *δ* at the corresponding surface, following the relation *α*=*kδ*=*kβL*, where *k*=2*π*/*λ*_0_ is the wave vector. In this manner, all of the ST slopes for the noise components produced in the stretcher and compressor can be calculated, and the results are listed in Table 1. Therein, *L*_1_ (*L*_2_) is the distance between the concave mirror and grating 1 (the image of grating 1). *L*_S_ is the distance between grating 1 and its image in the stretcher, and *L*_C_ is twice the distance between grating 2 and roof mirror 2 in the compressor. The slope *α* can be directly obtained from the positions of the noise peaks observed in the experiments.

## Author contributions

L.Q. and P.Y. conceived the original ideas. J.M., P.Y. and Y.W. designed and implemented the experiments. J.W. did the theoretical work. L.Q., P.Y. and H.Z. supervised the experiments and took part in the data analysis. All the authors contributed to the preparation of the manuscript.

## Additional information

**How to cite this article:** Ma, J. *et al*. Spatiotemporal noise characterization for chirped-pulse amplification systems. *Nat. Commun.* 6:6192 doi: 10.1038/ncomms7192 (2015).

## Figures and Tables

**Table 1:** Theoretical and measured ST noise slopes ***α***.

**Specifications**	**Concave mirror**		**Grating 1**	**Roof mirror 1**	**Roof mirror 2**	**Roof mirror 3**	**Grating 2**
	**B1&B4**	**B2&B3**					
Theoretical α*	*kβL*_1_	−*kβL*_2_	−*kβL*_S_	−*kβL*_S_	*kβL*_C_/2	*kβL*_C_	*kβL*_C_
Calculated *α*	6.9	−10.9	−4.0	−4.0	2.1	4.2	4.2
Measured *α*	5.2	−9.0	−3.8	−3.8	1.8	3.6	3.6
Mark in Fig. 3a							

^*^See Methods for the detailed theoretical analysis and the definition of each parameter. The unit of *α* is ps mrad^−1^.
